# Being Well Together: Individual Subjective and Community Wellbeing

**DOI:** 10.1007/s10902-019-00146-2

**Published:** 2019-07-13

**Authors:** Sarah Atkinson, Anne-Marie Bagnall, Rhiannon Corcoran, Jane South, Sarah Curtis

**Affiliations:** 1 Department of Geography and Institute for Medical Humanities, Durham University, Lower Mountjoy, Durham DH1 3LE, UK; 2 Centre for Health Promotion Research, School of Health and Community Studies, Leeds Beckett University, City Campus, Leeds LS1 3HE, UK; 3 Institute of Psychology, Health and Society, University of Liverpool, Waterhouse Building, Liverpool L69 3BX, UK

**Keywords:** Relationality, Inequality, Scale, Time, Settings, Culture

## Abstract

This paper explores the ways in which community wellbeing is, and could be, related to individual subjective wellbeing by mapping current practice, teasing out the assumptions underlying a dominant approach and flagging neglected issues. The notion of community is widely understood as about something more than the sum of the parts. Capturing subjective aspects of local life that are not simply individual but reflect the ways in which people feel and are well together is a challenging undertaking. Most existing frameworks for assessing community wellbeing are premised on a theory of the self as an autonomous, rational and independently acting or feeling individual, and the primary interest is on how community aspects of life impact on individual subjective wellbeing. This dominant approach consistently neglects spatial and social inequalities, multiple settings and scales and temporal choices and legacies, all of which constitute important political dimensions to community wellbeing. Social theories of the self as relational put relations as prior to subjectivity and as such afford ways to conceptualise community wellbeing in terms of being well together. A relational approach can also offer routes to tackling the complex interactions of inequality, scale and time. Such an approach is not, however, easily translated into quantitative measures or simple policy interventions. The approach taken to community wellbeing is not a technological issue but a political choice.

## 1 Introduction

The last decade has seen a renewed policy interest in asking what constitutes and shapes a good life. In the United Kingdom, political rhetoric on wellbeing and happiness has translated into new indicators to measure wellbeing, particularly subjective wellbeing. This is supported through a Centre on ‘What Works’ for Wellbeing established to summarise current knowledge across four broad areas including community wellbeing. Community wellbeing, however, is ‘a relatively new idea in social science’ and ‘it still lacks the theoretical structure for explanatory purposes’ (Sung and Phillips 2016, 2). Community is frequently conceptualised as an entity that is more than the sum of its parts and, as a social grouping, captures aspects of life as they are lived and experienced together (Sirgy 2011, 2018). This understanding demands a different approach from assessing individual or population wellbeing in terms of aggregated individual assessments. This paper explores the ways in which community wellbeing is, and could be, related to individual subjective wellbeing. We do this through an overview of current frameworks, a critical interrogation of their underpinning assumptions and their implications for policy focus, and a discussion of the potential for contemporary social theory to offer alternative starting points and approaches. The paper is based on three forms of information: frameworks widely referenced in the international literature and identified by searching google scholar for ‘community wellbeing’; a review of existing indicators for community wellbeing (Bagnall et al. 2016); critical reflection on both the dominant and alternative approaches informed by relational thinking in social and spatial theory.

The paper argues that the relationship between subjective and community wellbeing that is dominant in policy and practice is dependent on a particular, albeit implicit, understanding of the self. The first section maps existing prominent approaches to community wellbeing to draw out the underlying assumptions. A dominant framing of the relationship between subjective and community wellbeing shapes how policy attends selectively to certain aspects of community wellbeing. The second section elaborates this point through three areas of neglect in current approaches to community wellbeing: spatial and social inequalities; multiple settings and scales; temporal choices and legacies. The third section then explores how an approach to community wellbeing framed through relationality, rather than individual subjectivity, might address some of the existing policy omissions.

## 2 Subjective Wellbeing and Community: Current Approaches

### 2.1 Definitions

The concept of ‘community wellbeing’ comprises two terms, both of which are highly contested with no or little agreed consensus on their definition. Nonetheless, defining wellbeing has attracted an enormous amount of academic and policy attention, including in this journal, compared with the notion of community. This includes differentiating it from a series of affiliated concepts including quality of life, satisfaction, happiness or flourishing (Allin and Hand 2014). As a set, these concepts document the uptake of an argument very familiar to readers of this journal that public policy has tended to target the means, or determinants, of a good life, rather than the end, or ultimate policy goal, of a good life itself:

When we understand what makes people’s lives go well, see the positive things people bring to situations, and understand people’s emotional and social needs, projects and services can be better designed to respond to the many aspects that make up people’s lives. (NEF 2012, 8).

The recent increase in attention to subjective assessment of wellbeing is the logical end-point of this argument and acknowledges that only people themselves can report how they feel their lives are going. Nonetheless, the range of terms used in discussions of wellbeing, and the diverse understandings of each of these terms is confusing to inter-sectoral policy-making and may create a barrier to integrated decision-making and collective, joined-up action (Ereaut and Whiting 2008).

The concept of community also has a long history of debate about its meaning but this has received relatively little attention within the recent engagements with wellbeing (the launch in 2018 by Springer of the *International Journal of Community Wellbeing* is an important move towards redressing this). The conventional two-fold distinction, first made by Tönnies in 1887 (1957), between a community of residence (*gesellschaft*) and a community of shared values or interests (*gemeinschaft*) has been complemented by awareness of the many ‘communities’ within which any one person may enact their everyday lives (Orton et al. 2017). How a residential location intersects with multiple other ‘communities’ is an important consideration for policy making and recognised in the WHO’s attention to a settings-based approach to public health and wellbeing in the 1990s (WHO 1991). Nonetheless, at the turn of the Millennium, ‘community’ was still understood largely through these two major types, geographical and functional, and sharing the characteristic of people engaged in face-to-face communication, exchange and interaction (Fellin 2001). Since then, new forms of virtual and digital interaction, communication and relationship through the internet and social media have opened new spaces and potential expressions and interpretations of what a community can be. Moreover, the greater interconnectivity across different ‘scales’ from local to global that is captured under the general label of ‘globalisation’, makes clear that our understandings and experiences of ‘local’ and ‘community’ may no longer be easily fixed in territorial terms. These complex and multiple communities notwithstanding, Lee and Kim (2015) argue that the idea of community in relation to wellbeing remains usefully understood as a geographically bounded group of people at a local scale, usually residents in a locality, who are subject to direct or indirect interaction with one another. Contemporary governance is still organised and managed predominantly through the territorial jurisdictions of local authorities and, as such, policy often focuses on residentially defined communities. It remains the case, therefore, that in practice the dominant approach to community wellbeing draws on a territorial definition related to the neighbourhood and the local authority, urban or rural units and sub-national regions (e.g. ONS 2017).

Given the primacy of this territorial definition, community wellbeing effectively uses the term ‘community’ to qualify aspects of wellbeing that are of interest at the scale of a community as opposed to individual, national or international scales. National wellbeing is usually assessed through an aggregation of individual and territorial data for a selected set of domains. Thus, the UK measures of national wellbeing (ONS 2018) combine national information (e.g. inflation rate) with aggregated individual data (e.g. % reporting feeling happy yesterday). Nationally aggregated individual data provide measures of population wellbeing, in this case the population representing the nation-state. Community wellbeing, however, may aim to capture something rather different, although this depends importantly on the primary point of interest. If our interest is in how community scale factors impact on the individual wellbeing of the community’s members, then aggregating individual wellbeing scores is an appropriate approach. But if community is taken to be more than the sum of its parts then, as a social grouping, assessment needs to capture aspects of life, including wellbeing, as they are lived and experienced together (Howarth 2018; Sirgy 2018). Assessing wellbeing in terms of this collective aspect of life demands a different approach from assessing individual or aggregated population scale individual subjective wellbeing.

The paper’s aim, then, prompts the question of how community wellbeing is built from, or in relation to, subjective assessments. We distinguish three kinds of individual subjective assessment:

(a) subjective assessment of variables that affect individual lives (how we feel about our own house, our own job or our own levels of stress and happiness etc.);
(b) subjective assessments of variables that affect collective living (how we feel about local transport, the local economy or local safety, and local social factors such as level of trust in the community etc.);
(c) a third kind of assessment that is also, at least in part, subjective and that can capture community wellbeing as more than the sum of its parts, as being well together, is more challenging to define. Researchers have argued that assessments of community cohesion, shared values, belonging and ownership of community processes may reflect a collective mood that is a subjective form of community wellbeing (see Sirgy 2011; Bramston et al. 2002; Sung and Phillips 2018).

It is both useful and vital to keep sight of these different kinds and scales of indicators in order to be clear about what is being measured and what relationships might exist between these different scales of wellbeing.

### 2.2 Existing Frameworks

Given the apparent difficulties of pinning down the key attributes of community wellbeing, an alternative approach is to map how they are actually mobilised within policy and practice and to work backwards to identify the underlying premises and definitions. Bagnall et al. (2016) documented the variety of indicators for community wellbeing in use in the UK, and note that those explicitly using the language of community wellbeing are few (they report only five); they expanded their search to include closely affiliated terms. The 43 measures or indicators they identified describe a wide range of domains, although indicators for health and wellbeing, economy, services and infrastructure, environment and a range of variants on social association and inclusion were the most frequent. A parallel review of frameworks for variants of community wellbeing identified 27 different measurement tools but rated only five as excellent based on validity, reliability, responsiveness, length, use in cross-cultural settings, global scale assessment, inclusion of subjective measures, clarity and cost (Dronvelli and Thompson 2015). Moreover, only one, the Community Wellbeing Index used in Spain (Forjaz et al. 2011) offered a measure of local community based on individual assessments of the surrounding community. As such, the tool is useful for assessments of a community or of interventions that have their effects at the community level and so comes closest to capturing community wellbeing as collectively being well together that goes beyond the sum of the individual parts. Three other commonly referenced indicator sets are the OECD ‘How’s Life’ (2015), the Gallup Health Ways: Wellbeing Index (2019) and the Canadian index of Wellbeing (2013). These sets explicitly assess individual wellbeing and aggregate the data to construct territorially defined reports. Earlier work by Kusel and Doak (cited in Ribova 2000) in the Arctic regions of Canada included a concept of community capacity, which also features in the ‘Happy City Index’ (2019) (www.happycity.org) and in the Scottish Public Health Observatory’s ‘Place Standard’ (Scottish Public Health Observatory 2015).

Amongst the domains of community wellbeing, social relations is arguably the most important for capturing the sense of connectedness implied by the notion of community but is also the most problematic to translate into assessment. Concepts typically mobilised for this domain include: social networks, social support, social inclusion and exclusion, social cohesion, social capital, social justice, sense of belonging, sense of solidarity, respect and tolerance for diversity, gender equality, trust, reciprocity, security and safety, collaborative activities, local participation, political participation. The construct of social capital serves well to illustrate the complexities in mobilising such concepts.

Social capital has received a good deal of attention, including its uptake by the World Bank (Grootaert 1998). The seminal work argued that different forms of social association (weak, horizontal ties, bridging, bonding and linking forms etc.; Coleman 1990; Putnam 1993) constitute a resource for both collective groups of people and for individuals that can be conceptualised as capital. While much of the subsequent work arguably loses this key understanding of social association as a form of capital, there is a substantial body of evidence for the significance of social associations in managing the ups-and-downs of everyday life to the benefit of subjective wellbeing (Helliwell and Putnam 2004). Examples include availability and access to material and social resources (Bernard et al. 2007) or the kinds of spaces that facilitate building social capital and social cohesion, such as community organisations or public spaces in which people might run into one another informally (Cattell et al. 2008; Orton et al. 2017; Ross and Searle 2019). There remains, nonetheless, a tendency to focus on the benefits of social association for the individual rather than for collective groups, and, as such, it is important to remember that not all relationships constitute community or community wellbeing. The attention to social capital is also not without its critics. The networks of social association referenced by social capital may generate as much exclusion as inclusion (Portes 2014). Furthermore, capital is inherently social by virtue of the ways it is valued and distributed and to label only one aspect of everyday life as such may undermine this insight and thereby depoliticise associated social analyses (Fine 2010).

### 2.3 A Dominant Approach

The various frameworks for community wellbeing, despite variation and recognition of the conceptual and practical challenges involved, reveal a dominant approach in definition and operationalisation. This is characterised by the demands imposed by the need for assessment, conventionally through quantitative indicators, an ambivalence in the directionality of whether subjective wellbeing is the product or determinant of other aspects of a good life, and by a particular and pervasive understanding of the self in contemporary western political culture.

It is standard practice in policy-making to monitor progress in relation to the object of inquiry, in this case community wellbeing, and its possible determinants through quantifiable indicators of assessment. It is beyond the scope of this paper to engage the many critiques of such ‘calculative regimes’ beyond the widespread understanding of these as central to the practices of contemporary neoliberal governance (see Miller and Rose 2008). Existing approaches to community wellbeing follow a components approach (Atkinson et al. 2012) which accesses and makes it manageable by breaking it down into its component parts or domains. Assessment draws on a combination of objective and subjective indicators to monitor the community through aggregated individual attributes and descriptors of the territorial characteristics. There is variation in which domains are treated as components of community wellbeing itself and which as determinants of community wellbeing, depending on the particular focus of inquiry and mirroring similar variation with respect to individual wellbeing.

Subjective wellbeing has come increasingly to be presented as resulting from internal processes (e.g. mind-set, attitude, personality) rather than external influences and as influencing other levels of wellbeing including individual objective wellbeing (e.g. indicators of success). This reversed directionality, from the determinants of wellbeing to wellbeing as the determinant of other outcomes, takes its rationale from the positive psychology movement. While the importance of the social and of context are flagged (e.g. Seligman 2011), the core argument is that positive thinking and positive attitudes (e.g. optimism) can be learnt and taught and, in turn, impact on other aspects of individual wellbeing (e.g. www.actionforhappiness.org). The redirection of intellectual and popular attention to the inner self, rather than the external social context, may also be associated with a redirection of both private and public resources. In the more extreme versions of mindfulness, individual wellbeing derives from escaping the influence of the social altogether leaving any concern with community largely irrelevant (Whippman 2016). This shift to a self-help wellbeing may represent ‘new opportunities for human fulfilment, more (cost) effective policy impact through ‘behaviour change’, and more ‘people-centred’ policy’ or a ‘smokescreen for austerity or simple marketing ploy’ with ‘the potential to depoliticise by shifting attention from the level and quality of welfare provision to emotions and the self’ (White 2017, 121). Whichever it is, something important is happening here in terms of repositioning the place of the social and of the community. White (2017) describes a widespread cultural anxiety which she attributes to the erosion of value given to the social aspects of our lives. Whippman (2016) offers a similar critique, amassing substantial evidence on the importance of social life for human wellbeing alongside a critique of the isolationism characteristic of current popular inward-looking practices.

This dominant approach to community wellbeing is underpinned by the assumptions made about the nature of the self; these inform our interest in subjective wellbeing in the first place, how wellbeing is operationalised and where we locate the influences on our experiences of wellbeing. Approaches to individual subjective and community wellbeing build on a version of the self as a largely independent, autonomous and intentional individual. This characterisation of the self is documented in political theory as emergent with modernity and capitalism and entrenched within contemporary regimes of neoliberalism (Miller and Rose 2008). The growth of interest in the internal processes of mind, emotion and pre-cognition in recent years has deepened this way of thinking about our selves further, dubbed neuroliberalism (Whitehead et al. 2018). New and mobile technologies and methodologies can track our experiences moment-by-moment. These include the bio-sensing of physiological responses associated with emotions (see Aspinall et al. 2015), the analysis of social media posts (see Zeile et al. 2015), prompts for the immediate recording of experience through beepers (see Hurlburt 2017) or micro-phenomenological interviewing techniques (see Petitmengin et al. 2013). While this work has plenty of critics, advisories of caution and calls to combine methods (see Osborne and Jones 2017; Resch et al. 2015), these new approaches all give primacy to capturing the micro-changes and micro-temporalities of the inner self as the most authentic account of experience, emotion, cognition and our associated wellbeing.

This entrenching of an individually and internally defined self is, in turn, associated with a well-documented shift of emphasis in policy towards individual choice and responsibility for the care of our own wellbeing and those for whom we have caring duties (Sointu 2005) and away from concern with structural and social determinants, albeit not without resistance (Crawshaw 2012). Existing accounts of social ills as grounded in individual failings gain further backing through linking brain structure, anti-social behaviours and poor wellbeing (as positivity, self-esteem and so forth). This effectively reconfigures both poor individual wellbeing and inequalities in collective wellbeing as personal, rather than social or political, and social welfare policy reflects this in insisting on ‘attitudinal’ training for the individual management of wellbeing (Friedli and Stearn 2015).

This emphasis on the self as individually and internally constituted, as atomised, independent and autonomous, has two major implications for community wellbeing. First, it explains the relative absence of endeavours to capture the more-than-individual value that we might expect to be part of operationalising community wellbeing, and the support for understanding of wellbeing as primarily individual and of any population measure of wellbeing as properly represented by aggregated individual measures. Secondly, the embedding politics of individual responsibility translates into a similar shifting downwards of collective responsibility to local governance and civic organisations for supporting community wellbeing through local issues and strategies (Scott 2015). These two policy implications thus draw attention away from thinking about collective and community wellbeing as embedded in wider structures of politics and inequality and as shaped by factors operating across a range of scales and time. The next section explores some notable and surprising omissions in how the mainstream frameworks and indicators engage community wellbeing, many of which can be traced back to this dominant thinking about our individual self.

## 3 Neglected Aspects of Community Wellbeing

The existing frameworks for community wellbeing, based on dominant understandings of the self and of monitoring needs, lead to several important omissions in relation to considering community wellbeing. There are only two formulations that reference equality or equity (the Happy City and the University of Minnesota), only one (the Happy City) that references sustainability and almost no inclusion of cultural aspects, of what UNESCO terms ‘intangible cultural heritage’. As indicated at the end of the previous section, a focus on the individual and on local territories of residence and governance tends to prompt a parallel focus on determinants and processes operating at the local or individual scale. While there is research on the multi-scalar relations of wellbeing, this is certainly an area needing further attention (Schwanen and Wang 2014). There is a similar neglect of the multiple temporalities of wellbeing, involving the intimate flow of life-courses, inter-generational relations, processes of stability and sustainability, the longer trajectories of history, change and cultural heritage and the relationships between them. A specific consideration, one closely related to intangible cultural heritage and similarly lost in most schema, is any notion of a sense of place or community (Kee and Nam 2016) and of the histories of place that go beyond, or certainly deeper, than assessments of individual emotional attachments (Andrews et al. 2014; Gesler and Kearns 2002; Searle et al. 2009). These neglected aspects of community wellbeing involve a far greater focus on social and collective life, and an attention to our relations with the diverse processes and places that hinder or enable us to become well together.

### 3.1 Spatial and Social Inequalities

The omission of inequality seems particularly glaring as not only might it be included as an indicator in its own right, but there is an on-going debate about the importance of absolute and relative values for a range of material wellbeing indicators and their association with national wealth, local health and subjective wellbeing outcomes (Wilkinson and Pickett 2010). Moreover, the intersection between inequality and other aspects of community wellbeing is probably significant, given the social gradient in people’s participation in civic life. As such, it is crucial that assessment and intervention take account of both the historical and the current cultural context (Trickett et al. 2011). A further omission is any consideration of how a community may maintain and protect existing wellbeing, however defined. Frameworks predominantly focus on assessment and on the potential interventions to improve and grow wellbeing. This bias towards improvement overlooks the histories of post-industrial economic decline, environmental degradation or green belt housing developments, and population relocation schemes. These all attest to the processes through which community wellbeing is impacted by weakening sources of livelihood, bonding through employment networks, destruction of socially meaningful landscapes or beneficial greenspaces, or the scattering of established community groups to diverse locations. Local community strategies are vital for protecting and sustaining existing resources and opportunities whilst also addressing practices that may be discriminatory or harmful to certain sub-groups. In this, thinking about community wellbeing relates to the parallel conceptual and practical systems- and asset-based debate about what makes communities more ‘resilient’ (South et al. 2018).

Community wellbeing measures need to be amenable to disaggregation to socio-economic, demographic and sub-territorial levels in order to provide an additional community wellbeing measure of inequality across the territory. There is a debate here as to whether socio-economic or demographic groups really constitute ‘communities’ or whether these aggregated data might be more accurately termed measures of population sub-group wellbeing. Either way, the socio-economic and demographic categories identified within any society are likely to be highly significant groupings for documenting variations in collective wellbeing. Documenting variations in community wellbeing, however, is a very limited exercise. The important task is to interrogate and confront the processes and structural conditions of society producing differentiated and unfair inequalities in community wellbeing, or, as the Commission on Social Determinants of Health calls it, ‘the causes of the causes’ (Marmot 2007, 1153). A community characterised by inequalities is a community characterised by social injustice in the distribution of resources and opportunities. It is important, then, that statistics on the inequality in and between the wellbeing of territorial sub-groups is included as a key indicator of community wellbeing.

Socio-economic categories can be strongly associated with differentiated everyday experiences (OECD 2013) and capture the differentiated multiple positions and experiences within society, through which people’s identities are informed. Moreover, some constellations of these categories describe the most abject experiences in society which are often difficult to reach through surveys due to their relative invisibility (Tourangeau et al. 2014). This is an important point to emphasise; a community wellbeing measure that excludes, for example, trafficked and undocumented sex workers existing in most urban areas and who likely have extremely low wellbeing, or those without homes, fails to measure the contexts and practices facilitating such experiences. An awareness of the limitations of survey tools is important for at least two reasons. First, the size of a sub-population group living ‘below the radar’ will vary by territory. Comparisons of units of community wellbeing may be seriously misleading where communities differ in the size of their missing data. Secondly, while the invisibility of certain groups is unavoidable, their experiences are likely to reflect local inequalities that we can detect. Individual level assessments of individual and community scale domains can be aggregated to produce summary measures of sub-territorial groupings but indicators identifying sub-category or sub-territory information needs to be intentionally collected for this purpose. A community with good average wellbeing scores but which mask large sub-territorial inequalities does not align with most people’s idea of good community wellbeing.

### 3.2 Multiple Settings and Scales

The WHO settings approach, developed to advance health promotion (Dooris 2009), focuses on where and with whom people spend their time. In this approach, an individual can belong to multiple communities associated with different settings. This multiple communities approach has intuitive value for modern living: a person may, for example, be part of a residential community, a workplace community, leisure communities, online communities or even a homeless community. The issue for defining community wellbeing is whether to select just one of these multiple communities or whether to try to capture the more meaningful, but complex, range of belongings. If the individual’s wellbeing is assessed within different non- or only partially-overlapping settings, then there is no set of other individuals with whom to aggregate individual subjective wellbeing scores into a measure of community wellbeing. On the other hand, if an individual’s wellbeing is only aggregated with the other members of one bounded community (such as residence or workplace), much of the individual’s wellbeing may not be attributable to this single community. There is, therefore, a conceptual difficulty whichever way community wellbeing is approached. If, instead, community wellbeing is taken to primarily concern collective life as assessed through community level measures, then wellbeing inheres to the scale of the analysis, whether local, site or population group, and, as such, does not demand consideration of individual multiple settings. All of these options, however, do require consideration of the significance of different scales of analysis.

There is an important spatial consideration in conceptualising and assessing community wellbeing surrounding the decision about how to treat the different scales. This consideration concerns where to place those non-local wellbeing indicators, those aspects of life that do not strictly measure personal wellbeing but do describe the conditions that enable people to flourish. At the community level, local government and governance explicitly think in terms of indicators that inform ‘place-shaping’ policy and practice, ‘the creative use of powers and influence to promote the general wellbeing of a community and its citizens’ (Lyons 2000). This kind of approach moves beyond measures of community wellbeing based on compositional indicators generated by aggregating attributes of the individuals who make up communities to bring in contextual indicators which describe the wider determinants of wellbeing (Cummins et al. 2007). Depending on the nature of these wider determinants, they may be conceived as operating across local communities, regions, nations or even globally and represented as nested scales.

A multi-scalar approach demands explicit specification and justification of the population groups and scales of interest. A focus on community wellbeing tends to examine differences between neighbourhoods with findings that include subjective wellbeing as generally (although not consistently) lower in more densely populated, urban locations and countered by a tendency for wellbeing to be higher in populations with easier access to shops, schools, transport, health facilities and so forth. The inclusion of national scale factors into analysis indicates a reported tendency for deprivation, prosperity and resource availability, both at local and national levels, to influence local and individual subjective wellbeing (summarized in Schwanen and Wang 2014) and levels of wellbeing inequality (Abdallah et al. 2017; Curtis et al. 2017). There is, however, still relatively less exploration and comparison of factors associated with subjective individual and community wellbeing operating across the nation-state (Ballas and Dorling 2013). Moreover, the key determinants of subjective wellbeing vary across space and over time. Geographical research has initiatives to incorporate a longitudinal life-course perspective for places as well as people (Pearce 2018). Analysis of large European data-sets found that whether the absolute or the relative value in income and other indicators had greater influence on subjective wellbeing varied across different regions and countries, reflecting the importance of particular macro-political, economic and historical trajectories in any given setting (Aslam and Corrado 2012).

These studies reflect an approach of hierarchical scales, in which larger scales of analysis influence and shape smaller, or local, scales of analysis. A need to think about scale differently is evident where traditional values for community cohesion and unity are coming into tension with an emergent individualization of aspiration and consumption in the new economies of the growing peri-urban neighbourhoods of Latin America and Asia (e.g. Calestani 2012; Schaaf 2012). In this, the relationship between different scales is more complicated; trends at a global scale build from actions at the local scale but, in turn, the changes and tensions at the local scale reflect influences from the global scale. This demands an alternative multi-scalar analysis in which different scales are simultaneously interconnected and interacting in the production of wellbeing and of each other (Schwanen and Wang 2014). This sits intentionally in opposition to a conventional hierarchical approach in which the larger scale may influence and impact on the local but rarely vice versa (Marston et al. 2005).

### 3.3 Temporal Choices and Legacies

Very few schemes for community wellbeing explicitly include any conceptualisation of how community wellbeing may relate to time, which is strange given the avowed intent to monitor performance and progress over time.

An early engagement in the UK with the current renewed interest in wellbeing was led by the Department for Environment, Food and Rural Affairs (DEFRA). DEFRA explicitly asked whether wellbeing might serve as a useful concept in negotiating the tensions between policies for environmental sustainability and those for economic growth (NEF 2005). The importance of sustainability was thus at the heart of any consideration of wellbeing, and wellbeing in turn, was viewed as inseparably connected with the twin goals of a healthy future economy and a healthy future environment. Despite this early concern, current frameworks for wellbeing give little explicit attention either to sustainability or to the temporal frameworks within which wellbeing might be amenable to consolidation or change. The exceptions are the OECD framework for measuring wellbeing and the Happy City framework which both position sustainability as a primary dimension. The Happy City references sustainability as progress towards environmental goals for CO_2_ emissions, local recycling and energy consumption. The OECD framework references sustainability as the continued availability of key resources, viewed as forms of capital (social, human, natural and economic) which result from and in turn support community wellbeing in a continuous feedback loop.

The distinction made in the psychological literature between hedonic, pleasure-based, and eudaimonic, meaning and purpose-based, wellbeing is of note in this regard. Achieving an acceptable and adaptive level of wellbeing requires both forms. However, there is debate about how these relate to one another, how much of each is optimal, which is dominant and what the implications are of the different forms at individual level for wider considerations such as community wellbeing. Some have argued that eudaimonic wellbeing will always override the short-term gains of pleasure (for example, in Muirhead’s study of environmental volunteerism 2012). In contrast, psychologists describe a consistent and robust preference in human subjects for smaller, immediate rewards over larger, but deferred, rewards (Malkoc and Zauberman 2019). Social scientists argue that modern culture, characterised by the consumerism of contemporary capitalism, promotes and values hedonic wellbeing over the longer-term gains of meaning and purpose (Carlisle et al. 2012) with longer-term costs for sustainability of individuals, communities and, ultimately, the planet. The tensions between these two expressions of wellbeing play out locally, where local governments may favour ‘quick wins’ over longer-term strategies for lasting improvements. Planning for economic and environmental futures and the sustainable allocation of resources as the collective primary concerns requires that eudaimonic wellbeing through meaning and purpose become the individual primary concern. A eudaimonia-based policy approach, following Cresswell’s (2014) definition of places as spaces endowed with meaning, would explicitly aim to create places with purpose, where heritage, culture, industry and so forth define the actions of people in place and are associated with more resilient economies and environments. Foregrounding sustainability and other temporal processes draws attention to a range of local conflicts and interests in the allocation of resources and the benefits to community wellbeing. The Happy City framework recognises this by emphasising both sustainability and equality alongside the city conditions. How benefits to wellbeing are distributed and how this distribution changes over time is an important aspect of monitoring community wellbeing. Moreover, wellbeing gains for the community should not be at the cost of the wellbeing of future communities.

Inter-generational community wellbeing has received little direct attention, although debates in affiliated areas of social policy, such as employment, fees for higher education, pensions and, most recently, the Brexit referendum all reveal a major tension between the collective wellbeing of different age cohorts. Neglect of such tension is a serious omission in current work on community wellbeing. McGregor et al. (2000) describe the inter-generational contract for wellbeing:

‘In all ‘communities’…. there are relationships for the transfer of resources between generations and these relationships carry with them uncodified ‘rights’ and obligations… [we] … explore the transfers and processes governing transfers… heavy emphasis has been placed on the state in securing, if not actually institutionalising the inter-generational bargain. Wide ranging thinking and global social and economic forces require us to think more flexibly…and see [the bargain] as a more complex interplay of state, market, community and household.’ (McGregor et al. 2000, 447)

Work on inter-generational transfers tends to focus on material conditions and entitlements, the transmission of poverty from one generation to the next and the distributional inequalities of resource under austerity. It is, however, equally important to consider the transmission of non-material aspects of life, of meanings, values and relations, all of which contribute to how communities form their identity and self-define their collective wellbeing (Summer et al. 2009). Moreover, non-material dimensions of community wellbeing are essential components of the inter-generational transmission of material and bodily inequalities through both household and extra-household sites (Bird 2007). The centrality of shared non-material aspects and material resources in the inter-generational transmission of community wellbeing reaffirms the importance of a comprehensive approach to community wellbeing. These approaches, however, must also detect how wellbeing is differentiated by community sub-groups as well as between generations and have a longitudinal perspective that can both create and link together different sources of data. Designing this kind of study effectively is challenging and relatively few studies to date have done this compared with those using a cross-sectional design to identify associations and determinants of community wellbeing at any one time.

## 4 Relationality

Endeavours to include considerations of inequality, scale and time in understanding community wellbeing not only demand greater attention to community as greater than the sum of its parts but also afford routes into thinking about how to operationalise this. The conventional understanding of the individual as bounded, autonomous and existing outside of their social connections ignores a significant tranche of contemporary social theorisation on relationality. All schema for wellbeing, whether individual or community, always flag the importance of social relationships and relational entities such as trust or belonging, reciprocity, social integration or neighbourhood cohesion (Helliwell and Wang 2010; Uphoff et al. 2013). These, however, are most often only a resource for individual wellbeing, that is, as primarily instrumental to the independent, autonomously acting individual to realise their capacities or their potentialities. Relational theories reject the primacy, or even the pre-givenness, of the individual, the associated concepts of autonomy, rational choice or self-interest and the capture of these through individual data and statistical regressions. Instead, relations and interactions precede the definition of both individuals and collectives, of material things and immaterial values, of places and histories; relationality is inherent to who the individual is (see for example, Crossley 2011; Donati and Archer 2015; Gergen 2009). As White puts it, drawing on Gergen (2009), ‘This flips the switch, as it were, from seeing individuals as forging relationships, to viewing (multiple) relationships as forging individuals.’ (White 2017, 129). There are, however, important theoretical differences over the extent that being is always subsumed within relationality and whether non-relational processes, such as affect and corporeality, may sustain a residual singularity of being (see Gergen 2009; Harrison 2007).

Assessing relationality is challenging, which may in part explain its relative neglect in assessments of community wellbeing. Those that have tried position relationality as an intermediary between individual and community or collective scales.

Lee and Kim (2016) offer a pragmatic approach to consider relationality through a measure of inter-subjective community wellbeing. The concept of inter-subjectivity occupies a moderate position in relational theory. It describes the meanings each of us gives to our experiences and the knowledge we hold of the world as built individually through a set of senses and cognitions and inter-subjectively through our relations with others, mediated through our interactions, involving a reciprocity of perspectives and informed by our specific social and cultural reference points in the world (Anderson 2008). Inter-subjectivity also foregrounds a range of shared or public resources through which we make meanings, including concepts and language. Daniel Stern extended insights from his work on child development and inter-subjectivity to argue for an inter-subjective, narrative self (Stern 1998). Lee and Kim (2016) propose a distinction between satisfaction with (individual wellbeing) and evaluation of (inter-subjective community wellbeing) aspects of community life such as traffic conditions. Other surveys using questions that are evaluative could be conceptualised and analysed in this way. The UK ONS measures of national wellbeing, for example, include how safe people feel walking home at night, not just how satisfied they are with safety measures. Nonetheless, this mobilisation of inter-subjectivity still relies on the reports of the individual subjective respondent and the sense of an inter-subjective or relational identity remains elusive.

The emphasis on place-based approaches to community wellbeing can also enable a relational approach, through analysis of intersecting domains (Fleuret and Atkinson 2007; Winterton et al. 2014). White (2017) endorses this attention to the inter-dependency of different sites as strongly resonating with her empirically grounded field studies across countries in both the global south and north:

Wellbeing is understood as arising from the common life, the shared enterprise of living in community—in whatever sense—with others. Relationships thus form a central focus, as both the means through which (psychological, symbolic, social and material) goods are distributed and met, and as intrinsic to the constitution and experience of wellbeing. Subjective perceptions are anchored in material and relational contexts, producing a sense……of ‘life within limits’ (White 2017, 128).

Including relationality into thinking about subjective and community wellbeing brings to the fore issues of power and politics, as explicitly recognised and addressed by Prilleltensky (2008). He, too, posits the personal, the relational and the collective as three sites of wellbeing or, in his terminology, ‘wellness’, but emphasises how their inter-dependence demands attention to concerns of power, oppression and liberation (2008, 2012):

The third side of wellness concerns relational needs. Individual and group agendas are often in conflict. Indeed, like power, conflict is immanent in relationships. To achieve wellness, then, I claim that we have to attend to relationality as well. Two sets of needs are primordial in pursuing healthy relationships among individuals and groups: respect for diversity and collaboration and democratic participation. Respect for diversity ensures that people’s unique identities are affirmed by others, while democratic participation enables community members to have a say in decisions affecting their lives (Prilleltensky 2008, 122–123).

A more radical variant of relationality goes beyond social relationships in conceptualising how multiple relationalities not just with other people but also with structures, affects, materiality, places, other life forms and so forth, may combine to be intrinsically generative of identity, of stability, of change and of both individual and community wellbeing. The concept of the assemblage elaborates the coming together of diverse aspects of life in particular times and spaces such that all are equal participants (Delanda 2016; Deleuze and Guattari 1987). In this, each moment constitutes, and is constituted by, a particular assemblage and as such daily life is intrinsically unstable. However, multiple processes tend towards repetition, the repertoires of everyday habit and, as such, generate stability and predictability. The approach, however, allows for disruption, degeneration or transformation and the regeneration of new arrangements and habits for better or worse. Considerations of time within an assemblage includes historical trajectories and enables consideration of the ways in which inequalities are reproduced both structurally and affectively. Simple aggregations of individual subjective wellbeing routinely overlook these important considerations of historical and cultural contexts (Trickett et al. 2011). Whilst this complex approach is not easy to operationalise into a monitoring system, it does allow for multiple entry points at which intervention may shift, destabilise and reassemble the generative processes for individual or community wellbeing (Atkinson and Scott 2015). Research on assemblages relies on qualitative and ethnographic methods, as in the body of work to understand how places are therapeutic, restorative or enhancing in relation to wellbeing (Conradson 2005; Gesler 2003). Although this work has tended to focus on individual subjective wellbeing, the interaction with place is two way, relational and comprehensive (Duff, 2014) and as such affords an approach to a relational community wellbeing.

An alternative pathway to comprehending relationality is to engage the processes for defining the tools for monitoring as themselves contributing to local community wellbeing. The opportunity to set local criteria and local measures, at least in part, acknowledges the limited value, and feasibility, of resolving diverse engagements with community wellbeing into one single definition and framework. A preferable approach may be, instead, to build evidence of best practice about the processes of decision-making and a set of options for how community wellbeing is assessed (Warner and Kern 2013). There are examples of the processes through which local communities have defined their own measures of progress, whether from scratch or by selecting from an existing suite of indicators. Whilst the choice of measures that result may be little different from a set based on an existing framework, or defined by local authorities, the deliberation itself is important for community identity and wellbeing. Discussing and defining what is important locally serves to open discursive spaces as much as it results in a practical output (Scott and Bell 2013; Scott 2012). The transformative work involves promoting a participatory and democratic process, developing a set of conversations across the community about what is important and allowing, welcoming even, the identification and expression of conflicts of interest within a deliberative forum. Talking about community wellbeing itself becomes a means of exploration, understanding and developing local identity. For example, a focus on assets draws out the relational and material resources held in a community (Kretzmann and McKnight 1993). The stories that emerge, the narratives about place and history, both create local community and are accountable to the community’s visions of wellbeing. Critically the question of ‘what is a community asset’ varies by what is deemed of value by community members (Rippon and South 2017; South et al. 2017).

## 5 Individual Subjective and Community Wellbeing

Negotiating the multiple variants of definitions, measurement sets and, usually hidden, underpinning assumptions about being individual and collective can be a daunting task. A theoretical challenge remains, as perhaps it always has done (Allin and Hand 2017), with respect to conceptualising the complex relationships between interior life, self or relational selves and the external environment. Without explicit recognition of the assumptions made in operationalising these interactions, the pathways through which community level actions may impact on both community and individual levels of wellbeing remain similarly underspecified (Wakefield et al. 2001). We argue here that current practice in conceptualising and operationalising community wellbeing displays a dominant approach and that that this is underpinned by a particular understanding of the self as autonomous, rational and intentional. Theorisations of being that centre on relationality both enable a notion of community that is greater than the sum of its parts and foreground a series of neglected aspects in community wellbeing. Awareness of the different positions in relation to these complex inter-relationships is important as these come with different implications for policy and politics.

In thinking about how our lives go well in relation to other people, places, materiality and so forth, community wellbeing can be pragmatically defined through a set of domains of life that have meaning and importance locally but which are understood as imbricated within a wide range of interactions. This approach has two important policy variants: (1) a policy focus on how aspects of the local community impact on individual wellbeing, in which aggregated individual wellbeing, which is better understood as population wellbeing, becomes the key outcome measure; (2) a policy focus on the quality of collective life as a relational entity. The paper has foregrounded several critically important aspects to community wellbeing that current approaches almost entirely neglect: spatial and social inequalities; multiple settings and scales; temporal choices and legacies including sustainability and inter-generationality. It is our contention that thinking about community wellbeing premised on the autonomous, individual subject rather than attending to relationality not only results in an impoverished understanding of what it is to be human but, more significantly, results in obscuring the complex, enduring and iniquitous processes through which lives, individually and collectively, are unfairly differentiated.

The key issue in mobilising community wellbeing is, however, less which of the two policy options to choose but what balance to strike between them. This is not a technical question but a political question whose resolution will reflect different ideological positions about what it means to be human, how and at what scales living well is of interest, and where the most effective and politically acceptable entry-points are for intervention. We hope this paper prompts greater awareness and transparency about the positions that are taken in operationalising community wellbeing.
